# One-step refolding and purification of disulfide-containing proteins with a C-terminal MESNA thioester

**DOI:** 10.1186/1472-6750-8-76

**Published:** 2008-10-01

**Authors:** Maartje MC Bastings, Ingrid van Baal, EW Meijer, Maarten Merkx

**Affiliations:** 1 Laboratory of Chemical Biology, Department of Biomedical Engineering, Eindhoven University of Technology, P.O. Box 513, 5600 MB, Eindhoven, the Netherlands

## Abstract

**Background:**

Expression systems based on self-cleavable intein domains allow the generation of recombinant proteins with a C-terminal thioester. This uniquely reactive C-terminus can be used in native chemical ligation reactions to introduce synthetic groups or to immobilize proteins on surfaces and nanoparticles. Unfortunately, common refolding procedures for recombinant proteins that contain disulfide bonds do not preserve the thioester functionality and therefore novel refolding procedures need to be developed.

**Results:**

A novel redox buffer consisting of MESNA and diMESNA showed a refolding efficiency comparable to that of GSH/GSSG and prevented loss of the protein's thioester functionality. Moreover, introduction of the MESNA/diMESNA redox couple in the cleavage buffer allowed simultaneous on-column refolding of Ribonuclease A and intein-mediated cleavage to yield Ribonuclease A with a C-terminal MESNA-thioester. The C-terminal thioester was shown to be active in native chemical ligation.

**Conclusion:**

An efficient method was developed for the production of disulfide bond containing proteins with C-terminal thioesters. Introduction of a MESNA/diMESNA redox couple resulted in simultaneous on-column refolding, purification and thioester generation of the model protein Ribonuclease A.

## Background

Most common methods for protein bioconjugation rely on the reaction of amines to activated esters or thiol groups to maleimides. The limited control over the specificity of these reactions results in heterogeneous protein conjugates and frequently gives rise to protein inactivation. A variety of bio-orthogonal ligation methods that were originally developed in peptide chemistry have recently been applied to allow for controlled protein conjugation. Native chemical ligation (NCL) is a chemoselective reaction of a C-terminal thioester with an N-terminal cysteine to yield a native peptide bond under aqueous conditions. This method was originally developed by Dawson *et al*. in 1994 [1] to allow the chemical synthesis of proteins from smaller peptide fragments obtained by solid phase peptide synthesis [1-4]. The development of expression systems that use self-cleavable intein domains to generate recombinant proteins with a C-terminal thioester [5] resulted in a further extension of the possibilities for NCL. NCL has enabled the conjugation of recombinant proteins to spectroscopic labels, dendrimers, liposomes and micelles, as well as the site-specific immobilization of proteins to surfaces and the facile incorporation of unnatural amino acids [6-11]. Furthermore, posttranslational modifications that are inaccessible using standard *E. coli *expression systems can be introduced in recombinant proteins using NCL [12-14].

The C-terminal thioester that is required for NCL is easily obtained using the IMPACT™ system [5], which allows expression of the protein of interest as a fusion protein with a C-terminal intein domain followed by a chitin binding domain (CBD). Purification of the fusion protein using chitin affinity chromatography is typically followed by the addition of a small nucleophilic thiol, mercaptoethane sulphonic acid (MESNA). MESNA enables the cleavage of the target protein catalyzed by the intein domain, resulting in the production of a stable C-terminal thioester of the target protein. However, due to the reducing environment of the *E. coli *cytoplasm, disulfide bridges of recombinantly produced proteins are often not correctly formed. Refolding strategies for proteins with disulfide bonds typically use a glutathione redox buffer to assist in the refolding process [15-17]. We observed that incubation of proteins with a C-terminal MESNA – thioester with glutathione results in the formation of an unstable C-terminal glutathione-thioester, followed by hydrolysis to a carboxyl group.

Since the refolding procedure that is required to obtain disulfide containing proteins in their native state can lead to loss of the thioester functionality, NCL is typically performed directly on the immobilized fusion protein to yield a stable amide bond, followed by refolding in redox buffer systems [18,19]. However, this method does not allow precise control of important ligation conditions such as reaction stoichiometry and it cannot be used to generate stable thioester proteins for subsequent conjugation to cysteine-functionalized surfaces. The alternative to perform protein refolding after ligation of the unfolded protein to a surface is less attractive, since most protein folding procedures yield substantial amount of misfolded proteins that are then also attached to the surface or nanoparticle.

Here we report the development of a new redox buffer based on MESNA and its oxidized disulfide 2,2-dithiobis(ethanesulfonate) (diMESNA) that shows a refolding efficiency that is comparable to that of the glutathione redox couple. Using Ribonuclease A as model protein we show that inclusion of diMESNA during intein-mediated cleavage results in simultaneous on column refolding and thioester formation, providing a novel method for the production of correctly folded, disulfide-bridge containing proteins with a C-terminal thioester suitable for native chemical ligation.

## Results and discussion

### Novel MESNA/diMESNA redox couple

**Figure 1:**
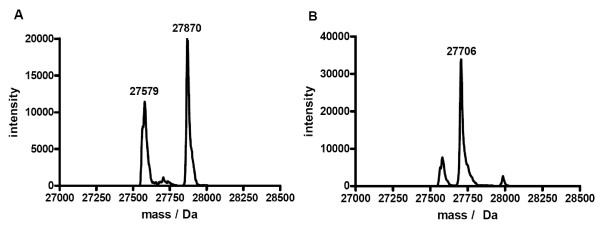
**GFP thioester exchange**. a) Deconvoluted mass spectrum obtained from ESI-MS analysis of GFP-MESNA incubated in glutathione buffer (3 mM GSH, 1 mM GSSG) for 10 h at RT. The peaks at 27870 Da and 27579 Da correspond to the glutathione-thioester of GFP (MW_calc _= 27869 Da) and hydrolyzed GFP with a carboxyl C-terminus (MW_calc _= 27576 Da), respectively. b) Deconvoluted mass spectrum obtained from ESI-MS analysis of GFP-MESNA incubated in MESNA refolding buffer (3 mM MESNA, 1 mM diMESNA) for 10 h at RT. The peak 27706 Da corresponds to the MESNA-thioester of GFP (MW_calc _= 27704 Da).

**Figure 2:**
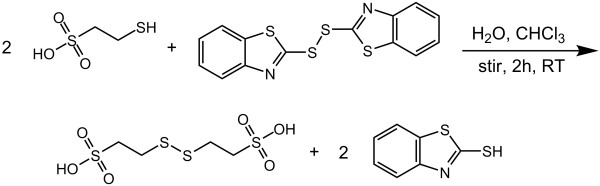
**Synthetic route to diMESNA**. diMESNA was synthesized according to literature procedures [20].

**Figure 3:**
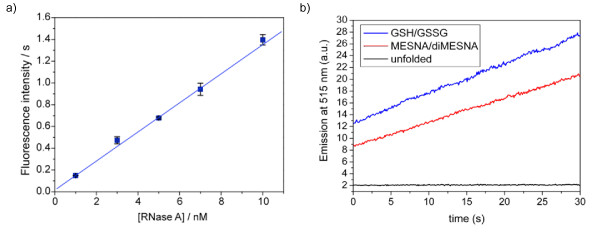
**Efficiency of the MESNA/diMESNA refolding buffer**. a) Calibration curve of the relation between initial enzyme activity and enzyme concentration. Error bars represent one standard deviation based on duplo experiments. All enzymatic activity measurements were performed in 0.1 M TRIS, 0.1 M NaCl, pH 8 buffer at 10°C with a substrate concentration of 400 nM. b) Enzyme activity of 5 nM unfolded RNase A and of 5 nM RNase A after refolding in either glutathione or MESNA refolding buffer (0.1 M Tris, 0.1 M NaCl, 3 mM reduced, 1 mM oxidized reagent; pH 8.0; 20°C, o/n); RNase A concentrations were calculated by comparison of the obtained initial velocity (from a linear fit to the first 30 seconds of the activity assay) to the calibration curve depicted in a).

The glutathione redox couple is the most common redox couple used to assist in protein refolding. To investigate the stability of protein thioesters in a glutathione redox buffer, green fluorescent protein (GFP) with C-terminal MESNA thioester was used as a test protein and incubated for 10 hours in glutathione refolding buffer. ESI-MS analysis showed significant transthioesterification and subsequent hydrolysis of the glutathione thioester (Figure 1a). To avoid the problem of transthioesterification with components of the redox buffer present in refolding buffers, we developed a MESNA-based redox buffer by synthesizing the oxidized, disulfide form of MESNA, 2,2-dithiobis(ethanesulfonate) (diMESNA) (Figure 2) [20]. Figure 1b shows that GFP-MESNA is stable in a MESNA-based redox buffer. A small peak corresponding to hydrolyzed GFP was observed after 10 h incubation at RT, but the amount of hydrolysis is significantly reduced compared to the incubation with glutathione redox buffer. The efficiency of the MESNA/diMESNA redox couple in protein refolding was tested using bovine pancreatic Ribonuclease A (RNase A). RNase A is a small protein of 124 amino acids that contains 4 disulfide bridges [21] and has been extensively used as a model to study the effects of disulfide bonds on protein refolding. Commercially available RNase A, purified by HPLC was unfolded in a 6 M guanidinium hydrochloride (Gu·HCl) denaturating buffer using tris(2-carboxyethyl)phosphine (TCEP) as reducing agent. Refolding was achieved by dilution of the unfolded RNase A in 3/1 mM MESNA/diMESNA containing refolding buffer. To compare this refolding buffer with conventional refolding conditions, refolding was also performed in a refolding buffer containing 3/1 mM GSH/GSSG. Refolding efficiencies were calculated based on an activity assay using the fluorescent 6-FAM-dArUdAdA-6-TAMRA substrate for RNase [22]. Kinetic measurements of the increase in FAM emission at 515 nm upon substrate cleavage by RNase A were performed. A linear fit to the initial 30 seconds of each activity assay yielded the enzyme's initial velocity. A calibration curve using known concentrations of commercial RNase A was used to determine the concentration of enzymatically active RNase A after refolding and thus the refolding efficiency (Figure 3a). Using this activity assay, refolding efficiencies of 58 ± 5% for the MESNA couple compared to 72 ± 5% of the glutathione couple after 24 hours of refolding were determined. These results show that the MESNA/diMESNA redox couple is a suitable alternative redox buffer for the refolding of RNase A (Figure 3b).

### On-column refolding

**Figure 4:**
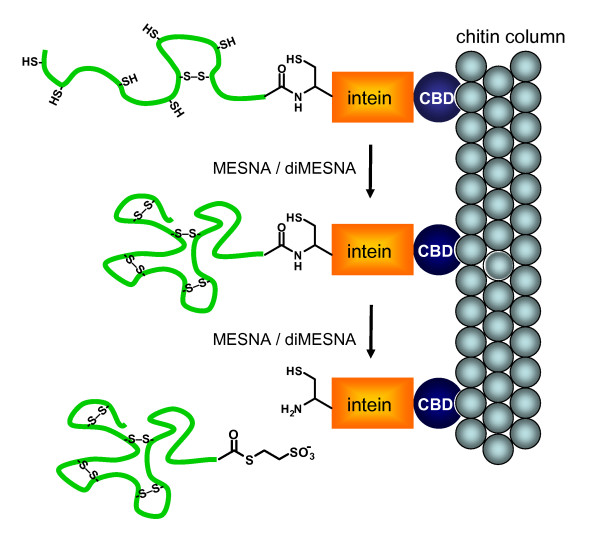
**On-column refolding and thioester generation**. Schematic representation of the simultaneous on-column refolding and thioester generation method. Following capture on a chitin resin incubation of RNase A – intein – CBD with MESNA and diMESNA results in simultaneous refolding of the RNase A and cleavage of the RNase A from the intein domain, generating a C-terminal MESNA thioester. The order of refolding and thioester generation is arbitrary.

**Figure 5:**
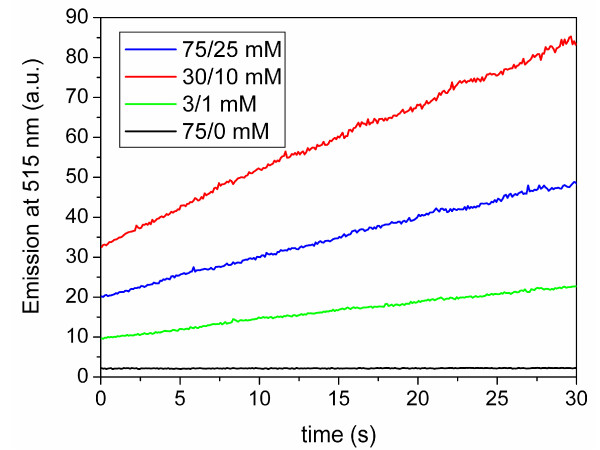
**Optimization of cleavage and refolding conditions**. Enzyme activity of cleaved RNase A-MESNA obtained after incubation for three days with different MESNA/diMESNA concentrations present in the cleavage buffer (50 mM MOPS, 500 mM NaCl, 0.1 mM EDTA, pH 6.0, at 20°C). Enzymatic activity measurements were performed in 0.1 M TRIS, 0.1 M NaCl, pH 8.0 buffer at 10°C with a substrate concentration of 400 nM.

We next tested whether protein refolding and MESNA-induced cleavage of immobilized RNase A-intein-CBD could be performed simultaneously (Figure 4). Raines and coworkers previously reported the expression of an RNase A-intein-CBD fusion protein in *E. coli *[18]. Because of the reducing environment of the *E. coli *cytoplasm, the RNase A domain was not correctly folded and inactive, but the fusion to the intein-CBD prevented its aggregation. The soluble fraction obtained after cell lysis that contained the RNase A-intein-CBD fusion protein was loaded onto a chitin column and washed to remove contaminating *E. coli *proteins. While keeping the ratio between MESNA and diMESNA constant at 3:1 (the ratio found in the cell's periplasm), three different concentrations of MESNA/diMESNA were tested, 3/1 mM, 30/10 mM and 75/25 mM. The highest RNase A activity was obtained with 30 mM MESNA and 10 mM diMESNA, which probably represents a compromise between thiol-induced cleavage, which is optimal at high concentrations, and the efficiency of refolding, which is optimal at low mM concentrations. As expected, incubation with only reduced MESNA (75 mM) yielded no active RNase A (Figure 5).

### Conjugation to Cys-PEG-DSPE

**Figure 6:**
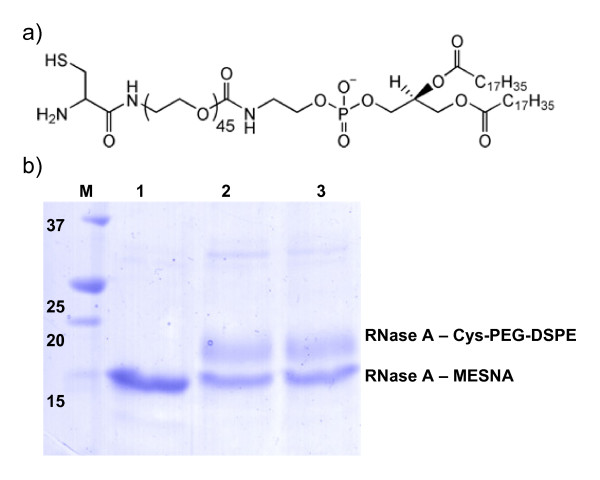
**Native chemical ligation of RNase A-MESNA to Cys-PEG-DSPE**. a) Structure formula of Cys-PEG-DSPE lipids. b) SDS-PAGE analysis of the reaction of refolded RNase A-MESNA (lane 1) (~10 μM) in HBS pH 7.4 with 50 mM MPAA and 1 mM Cys-PEG-DSPE (lane 2), or in HBS pH 7.4 with 50 mM MPAA, 10 mM TCEP and 1 mM Cys-PEG-DSPE (lane 3) at 20°C for 20 h.

Using this protocol of simultaneous column purification, refolding and thioester generation we routinely obtained 0.6 mg of active RNase A-MESNA per L of *E. coli *expression medium. To proof that the RNase A-MESNA was indeed active in NCL reactions we tested its reactivity with cysteine-functionalized pegylated phospholipids (Cys-PEG-DSPE). Cys-PEG-DSPE can be used to conjugate proteins in a controlled way to either form liposomes or micelles [7]. Overnight incubation of RNase A – MESNA with Cys-PEG-DSPE in a pH 7.4 HEPES buffered saline (HBS) and 50 mM 4-mercaptophenylacetic-acid (MPAA) as a catalyst resulted in a ligation yield of ~50% as estimated from the intensities of the bands of the SDS-PAGE gel. Moreover the absence of the reducing agent TCEP in the ligation reaction showed no negative effect on the ligation yield (Figure 6). These results show that the use of NCL to obtain well-defined protein liposomes and other cysteine-functionalized nanoparticles, can now be extended to disulfide containing protein domains.

## Conclusion

A novel procedure was developed that allows simultaneous purification, refolding and generation of a C-terminal thioester in recombinant proteins that contain disulfide bonds. The MESNA/diMESNA redox couple was shown to have a refolding efficiency that is comparable to that of the commonly used glutathione redox couple. The compatibility of the MESNA/diMESNA redox couple with the chitin column purification procedure for proteins with a C-terminal thioester was shown, resulting in on-column refolding simultaneously with cleavage of the protein from the intein domain and C-terminal thioester generation. This strategy should be generally applicable to generate disulfide-bond containing proteins with functional C-terminal thioesters for subsequent native chemical ligation.

## Methods

### General

Unless stated otherwise, all reagents and chemicals were obtained from commercial sources and used without further purification. Water was deionized prior to use. 6-FAM-dArUdAdA-6-TAMRA (Integrated DNA Technologies), 4-Mercaptophenylacetic-acid (MPAA) (Aldrich), Tris(2-carboxy-ethyl)phosphine hydrochloride (TCEP) (Sigma) were used as received. Ribonuclease A (grade XII-A, Sigma) was purified by reversed phase high pressure liquid chromatography (RP-HPLC) on a Varian Pro Star HPLC system coupled to a UV-Vis detector probing at 214 nm using a Vydac protein & peptide C18 column. A gradient of acetonitrile in water (both containing 0.1% trifluoroacetic acid (TFA)) was used to elute products. ESI-MS spectra were recorded on an Applied Biosystems Single Quadrupole Electrospray Ionization Mass Spectrometer API-150EX in positive or negative mode. Standard ^1^H NMR spectra were recorded at 25°C on a Varian Mercury 400 MHz spectrometer. Chemical shifts are given in ppm (δ) relative to tetramethylsilane (0 ppm). Fluorescence spectroscopy was performed on a Varian Cary Eclipse spectrometer at 10°C. All activity assays were performed in a 0.1 M Tris, 0.1 M NaCl, pH 8.0 buffer, with a substrate concentration of 400 nM.

### GFP thioester exchange

GFP-MESNA [6] (10 μM) was incubated in either glutathione refolding buffer (0.1 M Na-PO_4_, pH 8 containing 3 mM reduced glutathione and 1 mM oxidized glutathione) or MESNA refolding buffer (0.1 M Na-PO_4_, pH 8, containing 3 mM MESNA and 1 mM diMESNA) After 10 h incubation at RT, samples were characterized using LC-MS.

### Activity assay

RNase A (Sigma) was dissolved in assay buffer (0.1 M Tris, 0.1 M NaCl, pH 8.0) from a 40 μM stock. Upon addition of substrate (400 nM), fluorescence emission was monitored in time at 515 nm with excitation at 490 nm. The initial reaction rate v_0 _was calculated from a linear fit of the emission intensity in time over the first 30 seconds. A concentration range of RNase A was prepared and measured to obtain a calibration curve (1–10 nM; dilutions were freshly made before each measurement).

### Synthesis of 2,2-dithiobis(ethanesulfonate) (diMESNA)

MESNA (3.28 g, 0.02 mol) was dissolved in water (100 mL) and added to a solution of 2,2 dithiobis(benzothiozole) (3.32 g, 0.01 mol) in chloroform (250 mL). The reaction mixture was stirred vigorously for 2 hours at room temperature. After separation of the organic layer, the water was evaporated and the remaining white solid was dissolved in methanol/water 8:2 (150 mL). Acetone was added till the cloud point, and the product precipitated as a white solid during 5 days at room temperature. After filtering, a mixture of diMESNA and MESNA was obtained as a white powder in 70% yield (2.30 g, 8.2 mmol). ^1^H-NMR showed the presence of 25% of MESNA in the product. Since MESNA was added in all experiments further purification was unnecessary.

^1^H-NMR (400 MHz, MeOD) diMESNA δ = 3.2-3.1 (m, 4H, SO_3_CH_2_C*H*_2_SSC*H*_2_CH_2_SO_3_), 3.1-3.0 (m, 4H SO_3_C*H*_2_CH_2_SSCH_2_C*H*_2_SO_3_), MESNA δ = 2.9-2.8 (m, 2H, SO_3_CH_2_C*H*_2_S), 3.1-3.0 (m, 2H, SO_3_C*H*_2_CH_2_S)

LC-MS: m/z [C_4_H_10_O_6_S_4 _- H]- Calcd. 280.94 Da; Obsd 281.0 Da; [2M - H]- Calcd 562.87 Da Obsd. 562.7 Da

### Expression and purification of RNase A

The plasmid coding for RNase A-intein-CBD fusion protein, kindly provided by Prof. R. Raines (University of Wisconsin, Madison [18]), was transformed into chemically competent *E. coli *BL21(DE3) and plated on LB agar plates containing 100 mg L^-1 ^ampicillin. Single colonies were used to inoculate 2 mL LB medium containing 100 mg L^-1 ^ampicillin. Cultures were incubated overnight at 37°C and subsequently used to start 200 mL cultures containing 100 mg L^-1 ^ampicillin. At OD_600 _= 0.5 the temperature was lowered to 15°C and 0.3 mM IPTG was added to induce expression of the target protein. After overnight expression at 15°C and 250 rpm cells were collected by centrifugation, resuspended in BugBuster lysis buffer (Novagen) and incubated for 20 minutes at 20°C. A clarified cell extract was obtained by centrifugation at 40,000 ×g for 45 min. The supernatant was loaded onto a 10 mL chitin column (New England Biolabs) that was equilibrated with 20 mM 3-(N-Morpholino)-propanesulfonic acid (MOPS) NaOH, 0.5 M NaCl, 0.1 mM EDTA, pH 6.8 (column buffer). The column was washed with 10 volumes of column buffer to remove non- and weak binding proteins. Subsequently, 3 volumes of cleavage buffer (50 mM MOPS NaOH, 0.5 M NaCl, 0.1 mM EDTA, 50 mM MESNA, pH 6.0) were flushed quickly through the column. After overnight incubation of the column at 20°C, the MESNA thioester of RNase A was eluted from the column using 1 volume of cleavage buffer. SDS-PAGE analysis of the eluted protein showed a single band at ~14 kDa.

ESI-MS: deconvoluted mass (oxidized) Calcd. 13995 Da, Obsd. 14000 Da.

### Unfolding and refolding by dilution, glutathione vs MESNA redox couple

Unfolding: RNase A (0.465 mg, 33.9 nmol, SIGMA) was dissolved in 1 mL 4 M Gu·HCl, 100 mM TCEP. The mixture was incubated overnight at 20°C. Unfolding resulted in an increase of 8 Da from the 8 reduced cysteines.

ESI-MS: deconvoluted mass (reduced) Calcd. 13690 Da; Obsd. 13689 Da.

Refolding by dilution: Unfolded RNase A (500 μL) was added in 20 μL aliquots to 40 mL of refolding buffer (100 mM Tris·HCl, 100 mM NaCl; pH 8.0) containing either 3 mM reduced glutathione and 1 mM oxidized glutathione, or 3 mM MESNA and 1 mM diMESNA, and stirred overnight at room temperature. Refolding efficiencies were measured using the enzymatic activity assay at 5 nM initial RNase A concentration.

### On-column refolding and protein purification

A 10 mL chitin column was equilibrated with 10 volumes (100 mL) of column buffer. The column was loaded with cell extract and washed with 10 volumes (100 mL) of column buffer. Subsequently, 2.5 volumes of refolding/cleavage buffer (50 mM MOPS NaOH, 0.5 M NaCl, 0.1 mM EDTA, 30 mM MESNA, 10 mM diMESNA, pH 6.0) were flushed quickly through the column. The column was incubated for three days at room temperature and the product was eluted from the column with 10 volumes of cleavage buffer (50 mM MOPS NaOH, 0.5 M NaCl, 0.1 mM EDTA, pH 6). Fractions were analyzed by SDS-PAGE and enzyme activity assay, giving an estimated yield of 0.6 mg L^-1 ^active RNase A.

## Authors' contributions

MMCB developed the new refolding strategy and wrote the manuscript. IvB started the research and performed initial expression experiments. MM and EWM were responsible for the conceptual design of this project and contributed to the writing of the manuscript. All authors read and approved the final manuscript.
